# Treatment rationale and design of the induction chemotherapy and adjuvant thoracic radiation in resectable N2-3A/3B non-small cell lung cancer (ICAT) study

**DOI:** 10.1097/MD.0000000000016298

**Published:** 2019-07-05

**Authors:** Hiroaki Tsunezuka, Masayoshi Inoue

**Affiliations:** Division of Thoracic Surgery, Department of Surgery, Kyoto Prefectural University of Medicine, Graduate School of Medical Science, Kyoto, Japan.

**Keywords:** induction chemotherapy, postoperative radiotherapy, stage N2–3A/3B non-small cell lung cancer, trimodality therapy

## Abstract

**Background:**

The optimal treatment strategy for stage N2–3A/3B non-small cell lung cancer (NSCLC) remains controversial owing to its heterogeneity. Although multimodal therapy is considered the standard therapeutic approach for stage N2–3A/3B resectable NSCLC patients, the optimal combination strategy still needs to be clarified.

**Patients and methods:**

In total, 25 male and female patients aged between 20 and 75 years with stage N2–3A/3B resectable NSCLC will be included. Eligible patients will undergo tri-modality therapy comprising induction chemotherapy (3 cycles of combination therapy with carboplatin and nab-paclitaxel), followed by surgery and postoperative radiotherapy. Recruitment was commenced in April 2017, with a planned last follow-up in March 2024. As of May 2019, 1 subject has been enrolled. The primary endpoint is the treatment completion rate. The secondary endpoints are objective response rate (ORR) of induction chemotherapy, treatment-related adverse event, recurrence-free survival (RFS) time, and overall survival (OS) time. RFS and OS time will be calculated as the time from this study registration to first recurrence and all-cause death, respectively.

**Ethics and dissemination:**

The protocol was approved by the institutional review boards of Kyoto Prefectural University of Medicine and all the participating hospitals. Written informed consent was obtained from all patients before registration, in accordance with the Declaration of Helsinki. The study results will be disseminated via publication in peer-reviewed journals.

**Trial registration:**

Trial registration number UMIN000025010 and jRCT1051180028

## 1 Introduction

The optimal treatment strategy for stage N2–3A/3B non-small cell lung cancer (NSCLC) remains controversial due to disease heterogeneity. The PACIFIC study showed that concurrent chemoradiotherapy and adjuvant durvalumab therapy significantly increased the progression-free survival in patients with stage 3 unresectable NSCLC.^[1]^ Meanwhile, multimodality treatment with a combination of chemotherapy, surgery, and radiotherapy is considered the standard treatment option for stage N2–3A/3B resectable NSCLC. Patients who undergo surgery as part of trimodality therapy have better overall survival than those who undergo chemoradiotherapy alone,^[2]^ showing that surgery should be considered as part of a multimodality treatment for patients with resectable lung cancer. Induction chemotherapy combined with surgery could be beneficial and feasible for patients with resectable NSCLC.^[3–5]^ Furthermore, induction chemotherapy is non-inferior to induction chemoradiotherapy with respect to survival benefit.^[6,7]^ However, postoperative radiotherapy (PORT) improves survival and relapse.^[8–10]^ Stage N2–3A/3B NSCLC patients have high rates of distant metastasis and local recurrence, and aggressive consolidation therapy after induction chemotherapy and surgery improves survival benefit.^[11]^ However, some patients who undergo upfront surgery are ineligible for adjuvant chemotherapy because of a poor status after invasive surgery. More aggressive induction chemotherapy has higher feasibility and survival benefit and can ensure compliance to the planned chemotherapy. An effective combination approach will improve the feasibility and maximize the benefit of chemotherapy, surgery, and radiotherapy and improve survival. This study aims to evaluate the feasibility of trimodal therapy comprising chemotherapy, surgery, and radiotherapy for stage N2–3A/3B NSCLC. We will use a combination approach comprising 3 courses of induction chemotherapy, surgery, and PORT. We selected carboplatin (CBDCA) with nab-paclitaxel (PTX) as induction chemotherapy regimen. CBDCA with paclitaxel is one of the standard induction chemotherapy or chemoradiotherapy regimens for advanced NSCLC, and a phase III international trial reported that nab-paclitaxel (nab-PTX) regimen had a favorable risk-benefit profile compared with that of PTX because it improves the objective response rate (ORR) and decreases the risk of adverse events such as severe neuropathy and neutropenia.^[12]^

## 2 Materials and methods

### 2.1 Study design

**Figure 1:**
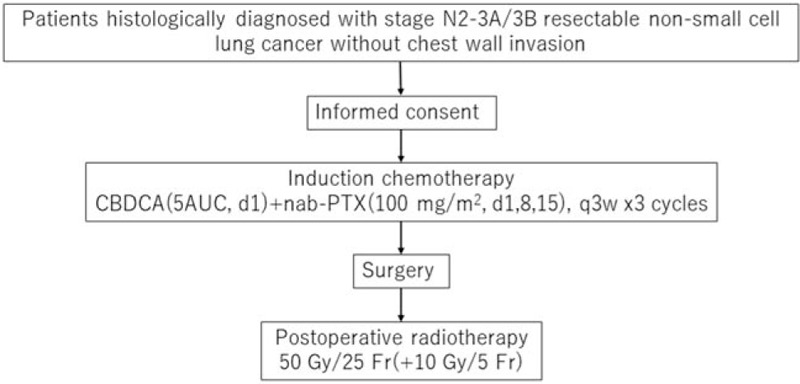
Study flow chart. AUC = area under the blood concentration curve, CBDCA = carboplatin, nab-PTX = nab-paclitaxel.

The study is an investigator-initiated, multi-institutional, single-arm, open-label prospective intervention phase II trial. Figure 1 depicts the study flowchart.

### 2.2 Study setting

Seven hospitals agreed to participate in this study. The protocol was approved by the institutional review board of each hospital. Written informed consent will be obtained from all patients before registration, in accordance with the Declaration of Helsinki. Patients will be registered in this study after independent review by the Center for Quality Assurance in Research and Development, Kyoto Prefectural University of Medicine. At least annual independent monitoring is planned, in accordance with the Japanese clinical trial guideline.

### 2.3 Participants

The tumors are staged according to the 8th edition of the Union for International Cancer Control TNM Classification of Malignant Tumors.^[13]^ Patients who were histologically diagnosed with N2–3A/3B NSCLC without chest wall invasion will be recruited.

The inclusion criteria are as follows:

(1) Complete resection after induction chemotherapy is anticipated.
(2) Confirmed normal respiratory function, which is defined as vital capacity (VC) >80% and forced expiratory volume in 1 second as percent of forced VC (FEV1%) >70%, within 56 days before obtaining informed consent.
(3) History of chemotherapy or radiation to the chest.
(4) Age between 20 and 75 years at the time of enrolment.
(5) Eastern Cooperative Oncology Group Performance Status of 0–1.
(6) Confirmed normal bone marrow, hepatic, and renal functions within 28 days before obtaining informed consent. The following clinical test standards were used:
  (1) Leukocyte count ≥3000/mm^3^
  (2) Hemoglobin ≥10.0 g/dL
  (3) Platelet count ≥100,000/mm^3^
  (4) Total bilirubin ≤1.5 mg/dL
  (5) Serum albumin ≥3.0 g/dL
  (6) Aspartate aminotransferase, alanine aminotransferase ≤100 IU/L
  (7) Creatine ≤1.2 mg/dL
  (8) Peripheral arterial oxygen saturation on room air ≥95%.
(7) Written informed consent.

The exclusion criteria are as follows:

(1) Unresectable mediastinal lymph node, such as extranodal infiltration, on computed tomography (CT)
(2) Having conditions contraindicated for CBDCA or nab-PTX administration
(3) Severe hypersensitivity to CBDCA or nab-PTX
(4) Severe bone marrow suppression
(5) Severe renal disorders
(6) Severe hepatic disorders
(7) Past history of severe drug allergy
(8) Pulmonary disorders
(9) Past history of cardiac infarction within 180 days before informed consent
(10) Chest CT showing possible interstitial pneumonia or idiopathic pulmonary fibrosis within 56 days before informed consent
(11) Prescription with steroids equivalent to >10 mg/day prednisolone within 90 days before informed consent
(12) Cardiac disorder of clinical concern
(13) Mental disorders of clinical concern
(14) Uncontrollable diabetes mellitus
(15) Infections of clinical concern
(16) Complications of clinical concern
(17) Active double cancer
(18) Patients who are pregnant, lactating, or potentially pregnant
(19) Any other patients regarded by the investigators as unsuitable for this study.

### 2.4 Dose and treatment regimens of induction chemotherapy

Induction chemotherapy will consist of 3 cycles of combination therapy with CBDCA (area under the blood concentration curve 5 mg/mL/min per Calvert formula) over 1 hour on day 1 and 100 mg/m^2^ nab-PTX over 30 minutes on days 1, 8, and 15 every 3 weeks. Dose modifications are allowed when grade 4 hematological toxicity or grade 2 to 3 nonhematological toxicity occur; toxicities are assessed according to the Common Terminology Criteria for Adverse Events (CTCAE) version 4.0.

### 2.5 Surgery

Clinical re-staging of the tumor after induction chemotherapy and before surgery will be performed via CT and positron emission tomography (PET)-CT. Tumor response will be evaluated according to the Response Evaluation Criteria in Solid Tumor (RECIST) version 1. Patients who achieve non-progressive disease status (PD, i.e., >20% increase in size or appearance of new lesions) will undergo surgery at 14 to 56 days after the end of induction chemotherapy. Surgical treatment will include lobectomy, bilobectomy, or pneumonectomy with systemic lymph node dissection. Pneumonectomy will be performed only when absolutely necessary. Open, minimally-invasive, or hybrid resection techniques are allowed in the trial.

Eligibility criteria for surgery:

(1) Complete resection after induction chemotherapy is anticipated
(2) Predicted postoperative (Ppo)% VC >40%
(3) Eastern Cooperative Oncology Group Performance Status of 0–1
(4) Confirmed normal bone marrow, hepatic, and renal function within 7 days before surgery according to the following parameters:
  (1) Leukocyte count ≥2,500/mm^3^
  (2) Hemoglobin ≥8.0 g/dL
  (3) Platelet count ≥70,000/mm^3^
  (4) Total bilirubin ≤1.5 mg/dL
  (5) Serum albumin ≥2.5 g/dL
  (6) Aspartate aminotransferase, alanine aminotransferase ≤100 IU/L
  (7) Creatine ≤2.0 mg/dL
  (8) Peripheral arterial oxygen saturation on room air ≥90%.

### 2.6 Postoperative radiotherapy

PORT will be performed within 56 days after surgery. Positive lymph node involvement on clinical imaging is defined as an enlargement of 1 cm or more in the short axis on CT scan and showing hypermetabolic uptake on fluorodeoxyglucose (FDG)-PET scan. The mediastinal target volume will be defined according to clinical guidelines and irradiated up to a total dose of 50 Gy in daily fractions of 2 Gy (Monday to Friday) within 56 days of PORT. In addition, subsequent boost irradiation of 10 Gy in daily fractions of 2 Gy will be applied in cases of positive margin (R1) or extracapsular tumor spread (total dose 60 Gy/30 Fr within 70 days of PORT). Three-dimensional CT images will be obtained for radiotherapy planning purposes. Opposing portal and multifield techniques are allowed in the trial according to irradiation methods. For pulmonary resection, we targeted an outcome of total lung V10 <40%, V15 <30%, and V20 <20%.

Eligibility criteria for radiation:

(1) Eastern Cooperative Oncology Group Performance Status of 0–1
(2) Confirmed normal bone marrow, hepatic, and renal function within 7 days before radiotherapy according to the following parameters:
  (1) Leukocyte count ≥2500/mm^3^
  (2) Hemoglobin ≥8.0 g/dL
  (3) Platelet count ≥70,000/mm^3^
  (4) Peripheral arterial oxygen saturation on room air ≥90%.
(3) The initially involved lymph nodes are documented quantitatively via CT scan, FDG-PET scan, and pathology.
(4) Complete resection during the surgery.

### 2.7 Rationale for setting the target population size

A total of 25 patients will be accrued in this study. Our analysis of previous trials showed that the complete resection rate after induction therapy was 71% to 76%.^[6,14]^ We expect a feasibility rate of >75% if this study used bimodality therapy with combination of induction chemotherapy and surgery without radiotherapy. However, because this will be a trimodality therapy study, the completion rate is estimated to be lower than that of bimodality therapy. The expected rate and threshold rate are determined to be 75% and 50%, respectively. Under these conditions, when 1-side alpha error of 0.1, beta error of 0.2, and statistical power of 80% are assumed, 21 subjects are required. Considering allowance for dropouts, the sample size was set to 25 patients.

### 2.8 Statistical methods

#### 2.8.1 Population to be analyzed

All subjects enrolled in this study (full analysis set, FAS), excluding the patients with serious violations (such as serious protocol deviation, violation for inclusion/exclusion criteria, and violation for prohibited concomitant medication/therapy) from FAS (per protocol set).

#### 2.8.2 Primary endpoint

The primary endpoint is the feasibility of trimodality therapy for resectable 3A/3B-N2 NSCLC, which will be evaluated according to the completion rate. Feasibility of trimodality therapy is defined as more than 50% of the enrolled patients completing the treatment. Assuming a null hypothesis of completion rate more than 50%, we will conclude that this trimodality therapy is useful. A 1-sided *P* value <.1 is considered statistically significant.

#### 2.8.3 Secondary endpoints

The secondary endpoints are ORR of induction chemotherapy, safety of the trimodality therapy, recurrence-free survival (RFS), and overall survival (OS). Both RFS and OS will be calculated as the time from this study registration to first recurrence and all-cause death, respectively

##### 2.8.3.1 ORR

Local response will be evaluated using images obtained via CT and PET-CT after induction chemotherapy in accordance with the RECIST guidelines.

##### 2.8.3.2 Safety: Treatment-related adverse event

The safety of chemotherapy and radiotherapy will be evaluated according to the CTCAE version 4.0, while the safety of surgery will be evaluated according to the Clavien-Dindo classification.

##### 2.8.3.3 RFS curve

Kaplan-Meier method will be used to estimate the RFS curve and calculate the annual and 2-year RFS and their 95% confidence interval.

##### 2.8.3.4 OS curve

Kaplan–Meier method will be used to estimate the OS curve, and the annual and 2-year OS rates and their 95% confidence interval will be calculated.

### 2.9 Ethics

The trial was approved by the Ethics Committee of Kyoto Prefectural University of Medicine, Kyoto, Japan (Approval number: ERB-C-765–2, the last edition ver 2 14/Feb/2018). The trial is subject to the supervision and management of the Ethics Committee.

### 2.10 Trial status

Recruitment was commenced in April 2017, with a planned last follow-up in March 2024. As of May 2019, 1 subject has been enrolled.

## 3 Discussion

The treatment of stage N2–3A/3B NSCLC remains controversial owing to its heterogeneity. Particularly, although multimodality therapy is considered the standard treatment option for stage N2–3A/3B resectable NSCLC patients, the optimal combination approach needs to be clarified. Chemotherapy is absolutely essential for the treatment of stage N2–3A/3B resectable NSCLC patients,^[3,5]^ while upfront surgery is associated with lower completion rate of adjuvant chemotherapy.^[15,16]^ Moreover, although there is no significant difference in survival benefit between neoadjuvant chemotherapy and adjuvant chemotherapy, but neoadjuvant chemotherapy is better tolerated as evidenced by the higher completion rate of full-dose chemotherapy and fewer high-grade toxicities.^[17]^ However, neoadjuvant chemotherapy increases postoperative complications and surgical complexity and thus requires careful surgical technique and postoperative management. Radiotherapy does not add any survival benefit to induction chemotherapy^[6]^ and increases the rate of severe postoperative complications, including bronchopleural fistula.^[6,18]^ A high completion rate will allow for maximized effect of multimodality therapy, particularly with respect to survival benefit, in stage N2–3A/3B resectable NSCLC patients. The ICAT study will provide data on the most appropriate combination approach of multimodality therapy for stage N2–3A/3B resectable NSCLC patients.

## Acknowledgments

We thank the patients, their families, and all investigators involved in this recent study.

Ikuya Inoue of the Center for Quality Assurance in Research and Development and Professor Satoshi Teramukai of Department of Biostatistics, Kyoto Prefectural University of Medicine, Graduate School of Medical Science are in charge of data management and analysis.

## Author contributions

**Conceptualization:** Masayoshi Inoue.

**Funding acquisition:** Hiroaki Tsunezuka, Masayoshi Inoue.

**Investigation:** Hiroaki Tsunezuka.

**Methodology:** Hiroaki Tsunezuka, Masayoshi Inoue.

**Project administration:** Hiroaki Tsunezuka, Masayoshi Inoue.

**Resources:** Hiroaki Tsunezuka, Masayoshi Inoue.

**Supervision:** Masayoshi Inoue.

**Visualization:** Hiroaki Tsunezuka, Masayoshi Inoue.

**Writing – original draft:** Hiroaki Tsunezuka.

**Writing – review & editing:** Masayoshi Inoue.

Hiroaki Tsunezuka orcid: 0000-0002-1101-8113.

## References

[1] Antonia SJ Villegas A Daniel D Durvalumab after chemoradiotherapy in stage III non-small-cell lung cancer. N Engl J Med 2017;377:1919–29. 28885881 10.1056/NEJMoa1709937

[2] McElnay PJ Choong A Jordan E Outcome of surgery versus radiotherapy after induction treatment in patients with N2 disease: systematic review and meta-analysis of randomised trials. Thorax 2015;70:764–8. 25967753 10.1136/thoraxjnl-2014-206292

[3] Rosell R Gomez-Codina J Camps C A randomized trial comparing preoperative chemotherapy plus surgery with surgery alone in patients with non-small-cell lung cancer. N Engl J Med 1994;330:153–8. 8043059 10.1056/NEJM199401203300301

[4] Scagliotti GV Pastorino U Vansteenkiste JF Randomized phase III study of surgery alone or surgery plus preoperative cisplatin and gemcitabine in stages IB to IIIA non-small-cell lung cancer. J Clin Oncol 2012;30:172–8. 22124104 10.1200/JCO.2010.33.7089

[5] NSCLC Meta-analysis Collaborative Group. Preoperative chemotherapy for non-small-cell lung cancer: a systematic review and meta-analysis of individual participant data. Lancet 2014;383:1561–71. 24576776 10.1016/S0140-6736(13)62159-5 PMC4022989

[6] Pless M Stupp R Ris HB Induction chemoradiation in stage IIIA/N2 non-small-cell lung cancer: a phase 3 randomised trial. Lancet 2015;386:1049–56. 26275735 10.1016/S0140-6736(15)60294-X

[7] Yang CF Gulack BC Gu L Adding radiation to induction chemotherapy does not improve survival of patients with operable clinical N2 non-small cell lung cancer. J Thorac Cardiovasc Surg 2015;150:1484–92. 26259994 10.1016/j.jtcvs.2015.06.062 PMC4651719

[8] Herskovic A Mauer E Christos P Role of postoperative radiotherapy in pathologic stage IIIA (N2) non-small cell lung cancer in a prospective nationwide oncology outcomes database. J Thorac Oncol 2017;12:302–13. 27746190 10.1016/j.jtho.2016.09.135

[9] Robinson CG Patel AP Bradley JD Postoperative radiotherapy for pathologic N2 non-small-cell lung cancer treated with adjuvant chemotherapy: a review of the National Cancer Data Base. J Clin Oncol 2015;33:870–6. 25667283 10.1200/JCO.2014.58.5380 PMC4348635

[10] Shen WY Ji J Zuo YS Comparison of efficacy for postoperative chemotherapy and concurrent radiochemotherapy in patients with IIIA-pN2 non-small cell lung cancer: an early closed randomized controlled trial. Radiother Oncol 2014;110:120–5. 24183868 10.1016/j.radonc.2013.10.008

[11] Amini A Correa AM Komaki R The role of consolidation therapy for stage III non-small cell lung cancer with persistent N2 disease after induction chemotherapy. Ann Thor Surg 2012;94:914–20. 10.1016/j.athoracsur.2012.04.088 PMC3468148 22819472

[12] Socinski MA Bondareko I Karaseva NA Weekly nab-paclitaxel in combination with carboplatin versus solvent-based paclitaxel plus carboplatin as first-line therapy in patients with advanced non-small cell lung cancer: final results of phase III trial. J Clin Oncol 2012;30:2055–62. 22547591 10.1200/JCO.2011.39.5848

[13] Eberhardt WE Mitchell A Crowley J The IASLC lung cancer staging project: proposals for the revision of the M descriptors in the forthcoming eighth edition of the TNM classification of lung cancer. J Thorac Oncol 2015;10:1515–22. 26536193 10.1097/JTO.0000000000000673

[14] Albain KS Swann RS Rusch VW Radiotherapy plus chemotherapy with or without surgical resection for stage III non-small-cell lung cancer: a phase III randomised controlled trial. Lancet 2009;374:379–86. 19632716 10.1016/S0140-6736(09)60737-6 PMC4407808

[15] Yoshino I Yoshida S Miyaoka E Surgical outcome of stage IIIA- cN2/pN2 non-small-cell lung cancer patients in Japanese lung cancer registry study in 2004. J Thorac Oncol 2012;7:850–5. 22481238 10.1097/JTO.0b013e31824c945b

[16] Zheng D Ye T Hu H Upfront surgery as first-line therapy in selected patients with stage IIIA non-small cell lung cancer. J Thorac Cardiovasc Surg 2018;155:1814–22. 29221745 10.1016/j.jtcvs.2017.10.075

[17] Brandt WS Yan W Zhou J Outcomes after neoadjuvant or adjuvant chemotherapy for cT2-4N0-1 non-small cell lung cancer: a propensity-matched analysis. J Thorac Cardiovasc Surg 2019;157:743–53. 30415902 10.1016/j.jtcvs.2018.09.098 PMC6344258

[18] Fujita S Katakami N Takahashi Y Postoperative complications after induction chemoradiotherapy in patients with non-small-cell lung cancer. Eur J Cardiothorac Surg 2006;29:896–901. 16675259 10.1016/j.ejcts.2006.03.023

